# Algorithm to Improve Resuscitation Outcomes in Patients With Traumatic Out-of-Hospital Cardiac Arrest

**DOI:** 10.7759/cureus.23194

**Published:** 2022-03-15

**Authors:** Hsin-Min Lee, Chia-Ti Wang, Chien-Chin Hsu, Kuo-Tai Chen

**Affiliations:** 1 Emergency Department, Chi-Mei Medical Center, Tainan, TWN

**Keywords:** major trauma, algorithm, resuscitation, outcome, traumatic out-of-hospital cardiac arrest

## Abstract

Background: This study proposed an algorithm to improve resuscitation outcomes in the emergency department (ED) for patients with traumatic out-of-hospital cardiac arrest (TOHCA). We also performed a retrospective chart review of patient outcomes before and after implementing the algorithm and sought to define factors that might influence patient outcomes.

Methods: In September 2018, we implemented an algorithm for patients with TOHCA. This algorithm rapidly identifies possible causes of TOHCA and recommends appropriate interventions. We retrospectively reviewed the outcomes of all patients with TOHCA during a five-year period (comprising periods before and after the algorithm) and compared the results before and after the implementation of the algorithm.

Results: After this algorithm was implemented, the use of the ED interventions of blood transfusion, placement of a large-bore central venous catheter, and thoracostomy increased significantly. The rate of return of spontaneous circulation (ROSC) also increased (before vs. after: ROSC: 23.6% vs. 41.5%, P = 0.035). Regarding hospital admission and survival to hospital discharge, we observed the trend of increment (hospital admission: 18.2% vs. 24.6%, P = 0.394; survival to hospital discharge: 0.0% vs. 4.6%, P = 0.107). Admitted patients exhibited a higher end-tidal CO_2_ level during resuscitation than nonadmitted patients [admitted vs. nonadmitted: 41.5 (33.3-52.0) vs. 12.0 (7.5-18.8), P = 0.001].

Conclusion: Our algorithm prioritizes the three major treatable causes of TOHCA: impedance of venous return, hypovolemia, and hypoxia. We found that rate of ROSC increased with the increasing implementation of the ED interventions recommended by the algorithm.

## Introduction

Advanced Cardiovascular Life Support is a clinical algorithm that guides the acute treatment of patients with cardiac arrest. A resuscitation team can employ this internationally accepted algorithm to make decisions efficiently under stressful conditions and provide consistent care for patients with nontraumatic out-of-hospital cardiac arrest [1]. However, a simplified and intuitive algorithm to inform the treatment of patients with traumatic out-of-hospital cardiac arrest (TOHCA) has yet to be developed [2,3]. Despite emergency physicians and trauma surgeons needing a standardized algorithm that can rapidly prioritize the various lifesaving interventions available to treat reversible causes of TOHCA.

In the study hospital, the resuscitation of a patient with cardiac arrest usually starts from standard Advanced Cardiovascular Life Support, which includes defibrillation for a shockable rhythm, continuous and uninterrupted chest compressions, emergent tracheal intubation with artificial ventilation, and injections of intravenous adrenaline every three to five minutes. If the patient develops a return of spontaneous circulation (ROSC), the resuscitation team will seek out any reversible causes of cardiac arrest and treat them if discovered. This approach is reasonable for patients with nontraumatic out-of-hospital cardiac arrest because lethal arrhythmia is the major reversible cause of cardiac arrest [1,2]. However, TOHCA has a different aetiology than nontraumatic out-of-hospital cardiac arrest [1-3]; using Advanced Cardiovascular Life Support may delay the timely treatment of reversible causes of TOHCA. Impedance of venous return, exsanguination, and hypovolemia are common causes of TOHCA [2,3]. Previous studies have demonstrated that external chest compressions are inadequate to maintain perfusion, which means patients with TOHCA may not benefit from standard Advanced Cardiovascular Life Support procedures [2-4]. Critical resuscitation time should be reserved for the treatment of reversible causes of cardiac arrest.

Accordingly, we designed a two-stage study for the resuscitation of patients with TOHCA. This study entailed the following aspects: proposing an algorithm to improve resuscitation in the emergency department (ED) for patients with TOHCA and performing a retrospective chart review of patient outcomes before and after algorithm implementation. The primary aim of this study was to clarify the effect of the algorithm. We also sought to define factors that might influence the outcomes of patients with TOHCA.

This article was published as a preprint in October 2021 (DOI: 10.21203/rs.3.rs-960250/v1).

## Materials and methods

Background and TOHCA treatment algorithm

The study hospital is a tertiary trauma center that admits approximately 2900 patients with trauma to the ED annually. When patients with trauma arrive at the ED, they are first examined and stabilized by emergency physicians (EPs) in the ED; trauma surgeons are involved later. Therefore, nearly all patients with TOHCA are resuscitated by EPs until the ROSC in the patient is achieved. In September 2018, we implemented an algorithm designed for caring for patients with TOHCA to assist EPs in making critical decisions within an extremely short window of time. Because penetrating injury is seldom encountered in East Asia [5,6], this algorithm is focused primarily on patients with blunt trauma. The fundamental aspect of this algorithm is rapidly identifying possible causes of cardiac arrest from clinical examination and the patient’s mechanism(s) of trauma and recommending interventions to correct them. We briefly introduce the content of the algorithm in the following paragraph.

(1) When a patient with TOHCA arrives at the ED, at least two EPs will participate in the resuscitation. We place the highest priority on three major treatable causes of TOHCA: impedance of venous return, hypovolemia, and hypoxia [2,4,7]. This algorithm encourages EPs to perform physical checks and use ultrasound to examine the patient for lesions that might impede venous return and evaluate the patient’s fluid status.

(2) If hemopneumothorax or pericardial effusion is suspected, bilateral thoracostomies and pericardiocentesis are performed as appropriate.

(3) Whenever hemorrhage is considered the cause of cardiac arrest, in addition to securing at least two peripheral intravenous routes and the rapid infusion of a 0.9% warm saline solution, the EPs set a large-bore (8.5F) central venous catheter (ARROW) via a femoral vein and begin a transfusion of 2-4 units of type O packed red blood cells. Any external bleeding and fractured proximal long bones are managed with direct compression and splinting.

(4) Next, the EPs perform tracheal intubation and commence mechanical ventilation to correct airway obstruction and hypoxia.

**Figure 1:**
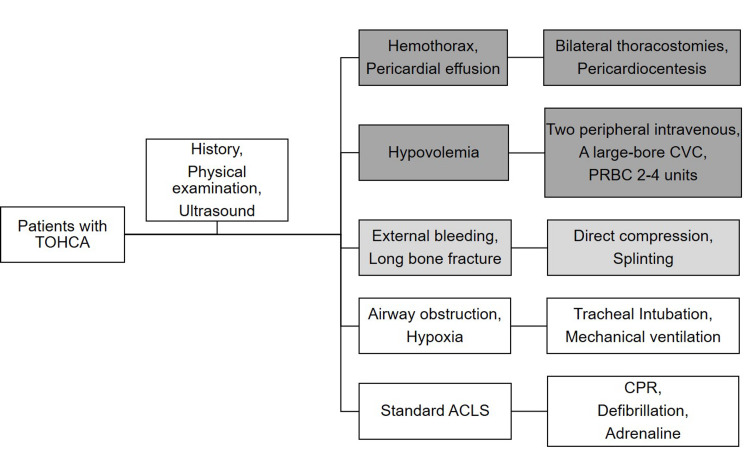
Algorithm flowchart. Blue blocks are priority interventions. ACLS: Advanced Cardiovascular Life Support CPR: cardiopulmonary resuscitation CVC: central venous catheter PRBC: packed red blood cell TOHCA: traumatic out-of-hospital cardiac arrest

Before the implementation of the algorithm, we held workshops and seminars about this algorithm and related videos on the algorithm on social media among the group of emergency physicians. Because EPs are familiar with all the required invasive procedures, this training focused on the concept of algorithms. This algorithm is not a mandatory requirement and we do not request all EPs followed the protocol during resuscitation. The flowchart and priority of interventions for this algorithm are displayed in Figure 1.

Data collection

We retrospectively reviewed the data of all patients with TOHCA who were admitted to the ED of the study hospital between 1 January 2016 and 31 October 2020. Data reviewed included age, sex, comorbidities, whether the patient’s collapse was witnessed, performance of bystander cardiopulmonary resuscitation (CPR), automatic external defibrillator deployment, prehospital defibrillation, prehospital airway management, prehospital CPR time, prehospital intravenous adrenaline injection, arresting rhythm, mechanism of injury, time from the scene to the hospital, initial level of end-tidal CO2 (ETCO2) derived from the patient’s endotracheal tube, CPR time in the ED, interventions (thoracostomy, pericardiocentesis, placement of central venous catheter, and blood transfusion) performed in the ED, and the presence of ROSC.

For patients with ROSC, we further examined data on the patient’s first cardiac rhythm after ROSC, pupillary light reflex, Glasgow Coma Scale (GCS) score after ROSC, time from CPR to ROSC, arterial blood gas analysis, lactate level, emergency surgery or transarterial embolization for bleeding, hospital courses, and neurological outcomes after hospital discharge.

Subgroup analysis: patients with exsanguination

Because this algorithm is specifically designed to improve the chance of survival for patients at risk of exsanguination, we excluded 12 patients whose presumed causes of death were hypoxia or arrhythmia (mechanisms of injury: hanging, drowning, asphyxia, and electrocution) and conducted a subgroup analysis for the remaining 108 cases. Rates of ED intervention, ROSC, hospital admission, and survival were tallied annually to calculate year-over-year changes.

The algorithm was evaluated and approved by the EPs conference. The Institutional Review Board of Human Research, Chi-Mei Medical Center, granted this study exemption from approval because the researchers used deidentified data. 

Statistical analysis

Descriptive statistics are provided as median and interquartile ranges (IQR) for continuous variables and as count and proportion for categorical variables. Statistical analyses were performed using SPSS 15 (SPSS, Inc., Chicago, IL, USA). We employed the chi-square test and Mann-Whitney U test to evaluate differences in dichotomous and continuous variables, respectively, between the various groups. Statistical significance was defined as P < 0.05.

## Results

Results overview

Of a total of 124 patients reviewed during the five-year period, we excluded four patients who had not undergone resuscitation because they were declared dead upon hospital arrival. Men constituted 66.6% of all patients and the median age was 53.5 years (IQR: 33.8-68.3). Overall, 40 patients (33.3%) achieved ROSC; 26 patients (21.7%) survived to hospital admission, and three patients (2.5%) survived to hospital discharge.

Prehospital data were only recorded for 109 patients; data were lost for 11 patients. Fifty patients’ (45.9%) collapses were witnessed by others, and 27 patients (24.8%) received bystander CPR. The median time from the scene to the hospital was 21.0 min (18.0-26.8) and prehospital CPR time was 11.0 min (6.0-16.0). Fifty-seven patients (52.3%) underwent prehospital airway placement, including 56 patients with supraglottic airway and one patient with endotracheal tube. Automatic external defibrillators were deployed for 68 patients (62.4%), and among these patients, defibrillation was delivered to six patients (5.5%). Six patients (5.5%) underwent intravenous prehospital adrenaline injection. The arresting rhythms were as follows: 60 patients (55.0%) with pulseless electrical activity and 49 patients (45.0%) with asystole.

**Figure 2:**
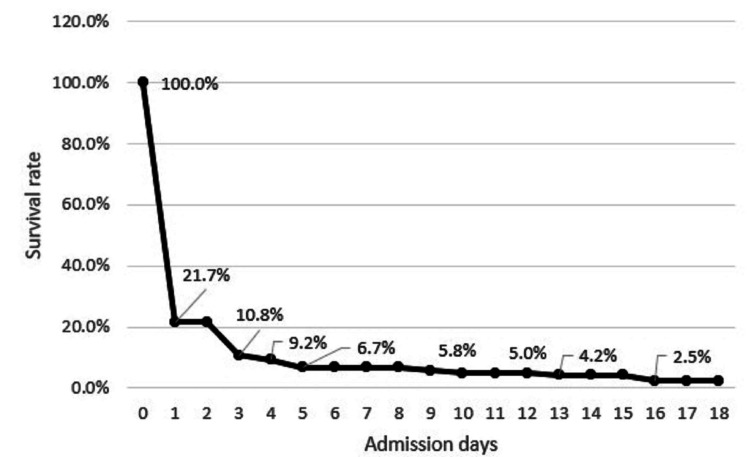
Survival curve of patients with traumatic out-of-hospital cardiac arrest (TOHCA).

The mechanisms of trauma were traffic accident (60.8%), fall (19.2%), hanging/drowning/asphyxia (13.3%), penetrating injuries (2.5%), and others (4.2%). The median time of CPR in the ED was 26.5 min (IQR: 10.8-33.3). The number and occurrence of ED interventions are listed below: transfusion (50, 41.7%), placement of a large-bore central venous catheter (36, 30.0%), thoracostomy (21, 17.5%), and pericardiocentesis (two, 1.7%). ETCO2 levels were recorded in 60 patients, and the median level was 7.0 mmHg (IQR: 1.0-24.0). Forty patients (33.3%) achieved ROSC. However, 14 patients did not survive long enough in the ED and only 26 patients (21.7%) were admitted to the intensive care unit. Among the admitted patients, one patient died and became an organ donor after 15 days of intensive care, three patients survived to hospital discharge, and only one patient recovered to normal daily activity (he can conduct regular activity and resume to work). The survival curve is plotted in Figure 2.

**Figure 3:**
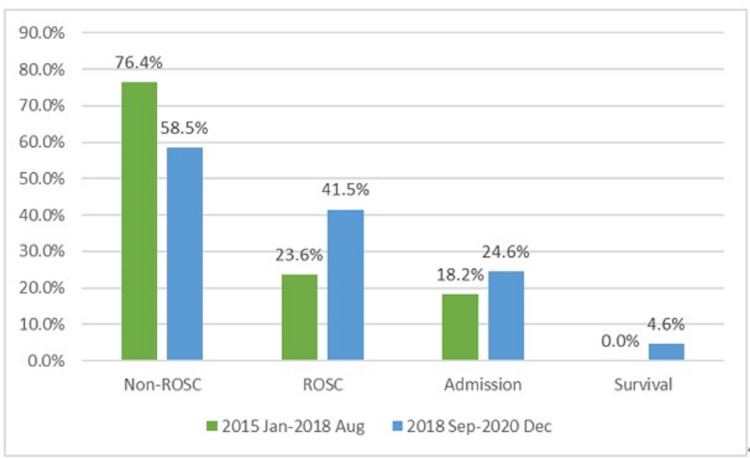
Rates of ROSC, hospital admission, and survival increased after the algorithm was implemented. Among the patient with ROSC, a significant proportion of patients did not survive long enough to admit to intensive care unit. Non-ROSC: non-return of spontaneous circulation ROSC: return of spontaneous circulation

Figure 3 shows that rate of ROSC increased after the implementation of algorithm (before vs. after: ROSC: 23.6% vs. 41.5%, P = 0.035). Regarding hospital admission and survival to hospital discharge, we also observed the trend of increment (hospital admission: 18.2% vs. 24.6%, P = 0.394; survival to hospital discharge: 0.0% vs. 4.6%, P = 0.107).

Comparison between ROSC and non-ROSC

Clinical variables for patients with and without ROSC are presented in Table 1 for comparison. Neither group had statistically significant differences in sex, age, or comorbidities. Patients with ROSC had lower rates of automated external defibrillator deployment and pulseless electrical activity at ED arrival than patients without ROSC; otherwise, prehospital data were similar for both groups. Patients with ROSC had an insignificantly higher rate of hanging/drowning/asphyxia than patients without ROSC (17.5% vs. 11.1%, P = 0.342). In ED management, patients with ROSC had a shorter CPR time, higher ETCO2 level during resuscitation, and a higher occurrence of large-bore central catheter placement and blood transfusion.

**Table 1:** Demographics, prehospital data, mechanisms of trauma, and emergency department interventions in the return of spontaneous circulation (ROSC) and non-ROSC groups. The data are provided as median and interquartile range for continuous variables and as proportions for categorical variables. CPR: cardiopulmonary resuscitation AED: automatic external defibrillator PEA: pulseless electrical activity ETCO2 level: level of end-tidal carbon dioxide

	ROSC	Non-ROSC	p
Age (year)	55.0 (28.0-66.0)	51.0 (35.8-71.3)	0.434
Sex (male)	62.5%	68.8%	0.494
Comorbidity	17.5%	25.0%	0.354
Prehospital data			
Witness collapse	54.5%	42.1%	0.231
Bystander CPR	33.3%	21.1%	0.172
Deployment of AED	45.5%	69.7%	0.016
Defibrillation	3.0%	6.6%	0.455
Prehospital time (min)	22.0 (19.0-27.0)	21.0 (18.0-26.3)	0.526
Prehospital airway	39.4%	57.9%	0.076
Prehospital CPR (min)	9.0 (3.0-16.0)	12.0 (7.8-16.0)	0.250
Prehospital epinephrine	9.1%	3.9%	0.279
Arresting rhythm: asystole	61.8%	6.1%	< 0.001
Arresting rhythm: PEA	38.2%	93.9%	< 0.001
Mechanisms of trauma			
Traffic accident	62.5%	60.0%	0.791
Fall	20.0%	20.0%	1.000
Hanging/drowning/asphyxia	17.5%	11.3%	0.342
Penetrating injuries	0.0%	3.8%	0.215
Others	0.0%	6.3%	0.106
Emergency department data			
CPR time	9.0 (5.0-16.0)	30.0 (25.0-35.0)	< 0.001
Central venous catheter	55.0%	17.5%	< 0.001
Thoracostomy	17.5%	17.5%	1.000
Blood product transfusion	67.5%	28.8%	< 0.001
Pericardiocentesis	0.0%	2.5%	0.313
ETCO2 level	34.0 (15.0-49.5)	7.0 (2.0-11.0)	< 0.001

After achieving ROSC, cardiac rhythms were sinus tachycardia (55.0%), normal sinus rhythm (37.5%), and atrial fibrillation (7.5%). Four patients with exsanguination required invasive treatment, namely two abdominal surgeries and two transarterial embolizations. Cranial surgery was performed in one patient due to a severe traumatic brain injury. Only three patients exhibited a positive pupillary light reflex after ROSC; two of these patients had eye and motor GCS scores above 1. Both patients with a positive pupillary light reflex and elevated GCS scores survived, and one patient recovered to normal daily activity.

**Figure 4:**
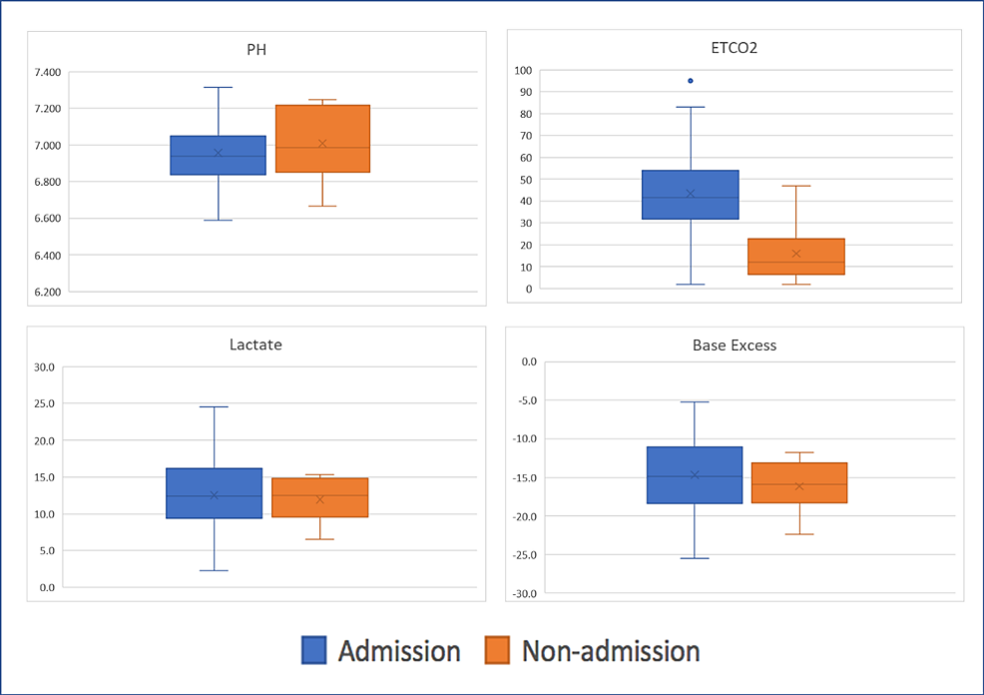
Comparisons between the hospital admission group and nonadmission group; levels of end-tidal CO2 in the admission group were higher than in the nonadmission group; the groups exhibited statistically equivalent results in pH, base excess, and lactate levels. ETCO2: level of end-tidal carbon dioxide

A comparison of laboratory test data between admitted and nonadmitted patients is presented in Figure 4. Admitted patients exhibited higher levels of ETCO2 than nonadmitted patients [admitted vs. nonadmitted: 41.5 (33.3-52.0) vs. 12.0 (7.5-18.8), P = 0.001]. Both groups exhibited statistically equivalent results in potential of hydrogen (pH), base excess, and lactate levels [admitted vs. nonadmitted: pH: 6.938 (6.843-7.044) vs. 6.987 (6.871-7.216), P = 0.571; base excess: 14.6 mmol/l (11.4-18.4) vs. 15.6 mmol/l (13.1-17.8), P = 0.655; lactate: 12.4 mmol/l (10.2-14.4) vs. 10.7 mmol/l (8.6-14.2), P = 0.773].

Exsanguination-related TOHCA

**Figure 5:**
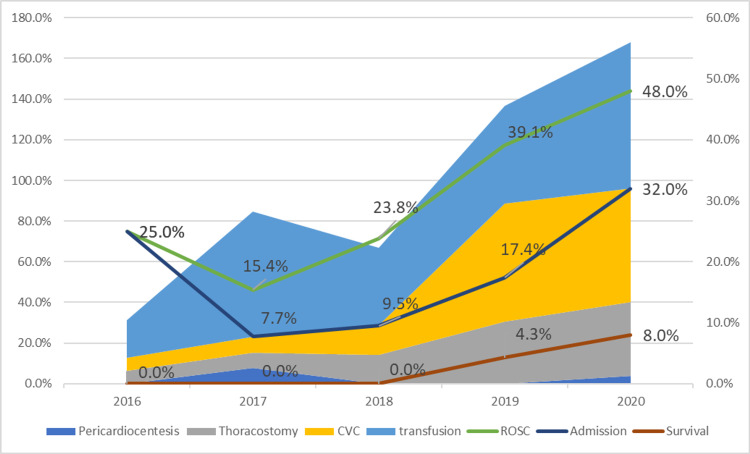
Since the implementation of this algorithm in September 2018, ED interventions of blood transfusion, large-bore CVC, and thoracostomy increased. Additionally, we observed the trend of increments in rate of ROSC, hospital admission, and survival to hospital discharge. CVC: central venous catheter ED: emergency department ROSC: return of spontaneous circulation

For patients with suspected exsanguination-related TOHCA, the annual rates of ROSC, hospital admission, and survival to hospital discharge from 2016 to 2018 were poor (2016: ROSC: 25.0%, hospital admission: 15.4%, survival to hospital discharge: 23.8%; 2018: ROSC: 20.5%, hospital admission: 7.7%, survival to hospital discharge: 9.5%, respectively). None of the patients survived to hospital discharge. After the implementation of this algorithm, the ED interventions of blood transfusion, placement of a large-bore central venous catheter, and thoracostomy increased significantly. The rates of ROSC, hospital admission, and survival to hospital discharge increased to 39.1%, 17.4%, and 4.3% in 2019 and 48.0%, 32.0%, and 8.0% in 2020. Figure 5 displays an increase in rates of ED interventions and rates of ROSC, hospital admission, and survival to hospital discharge for patients with exsanguination.

## Discussion

Severe head injury and hypovolemia account for approximately 90% of TOHCA. Other common aetiologies include hypoxia, tension pneumothorax, pericardial tamponade, and injuries to the heart and major blood vessels [2,4,8]. Among these aetiologies, patients with severe injuries to vital organs such as the brain, heart, and great vessels can seldom be resuscitated [2,9]. However, resuscitation teams may be able to correct hypoxia, hypovolemia, and impedance to venous return-for instance, pneumothorax or pericardial tamponade. The algorithm presented herein is has an operating principle comparable to that of two published algorithms which emphasize the early recognition and treatment of reversible causes of TOHCA [4,7]. The algorithm demonstrated beneficial effects in this study. According to published data, the overall survival rate of TOHCA is approximately 5.6% [8]. Before implementing this algorithm, the survival rate for patients with TOHCA in the study hospital was 0.0%. The survival rate increased to 4.6% after implementing the algorithm and reached 6.6% in the final year of the study.

In the resuscitation of citizens and soldiers, obtaining vascular access and rapidly infusing blood and crystalloid fluid are paramount resuscitative measurements [10,11]. This study demonstrated that the rate of ROSC increased with the placement of a large-bore central venous catheter and blood transfusion. The restoration of intravenous fluid results in more cardiac output from chest compressions and hence a better chance of ROSC.

We did not find that patients with ROSC underwent thoracostomy more often than patients without ROSC. In patients with exsanguination, an increase in thoracostomy was correlated with an increase in ROSC, hospital admission, and survival. Early thoracostomy is thought to be associated with better outcomes in patients with TOHCA because hemopneumothorax relief improves venous return, which increases cardiac output during resuscitation.

In cases of nontraumatic out-of-hospital cardiac arrest, prehospital arresting rhythm, defibrillation, airway management, and intravenous adrenaline have been reported to be key prognostic factors for ROSC [1,6,12-14]. However, these factors were not correlated with higher rates of ROSC for patients with TOHCA in this study.

Only 1.6% and 1.5% of patients with TOHCA in Australia and Taiwan, respectively, presented with a shockable rhythm [6,15]. In this study, both automatic external defibrillator deployment and prehospital defibrillation failed to correlate with a higher rate of ROSC; patients with a shockable rhythm accounted for 5.5% of the total study cohort. Accordingly, we conclude that analysis of the arresting rhythm and defibrillation provide a minimal beneficial effect in patients with TOHCA.

A systemic review evaluated the benefits and harms of three airway management approaches (bag valve mask, supraglottic airway, and endotracheal intubation) used in prehospital settings on patients with TOHCA [13]. The review did not demonstrate that more invasive airway approaches improve survival and ROSC rates. A prospective, multicenter, observational study conducted in Japan demonstrated that the use of adrenaline during prehospital resuscitation decreased the survival rate of patients with TOHCA [14]. In this study, patients with ROSC did not present with a statistically significantly higher rate of prehospital insertion of airway devices and intravenous adrenaline than patients without ROSC. Consequently, neither our literature review nor the current study supports the use of prehospital invasive airway devices and adrenaline in patients with TOHCA. Perhaps ‘load and go’ is still the best strategy for patients with out-of-hospital cardiac arrest if a short transportation time is anticipated.

For patients who achieved ROSC, we could not draw conclusions about successful hospital admissions based on levels of PH, base excess, or lactate. Only a high ETCO2 value was associated with a higher rate of hospital admission. Bulgar et al. studied the relationship between prehospital ETCO2 values and haemorrhagic shock in intubated trauma patients [16]. Patients in haemorrhagic shock had lower ETCO2 values than those not in haemorrhagic shock. We took ETCO2 values after ROSC to represent the severity of shock in patients with TOHCA. Moreover, this finding further supports the initial assumption of the algorithm that the faster the causes of shock or death can be reversed, the better patient outcomes will be.

When to terminate resuscitation of patients with TOHCA is controversial. Early guidelines stipulated that resuscitation efforts should be terminated after 15 min of unsuccessful resuscitation [17]. However, with the improvement of resuscitation algorithms and knowledge about TOHCA, the overall survival rate from TOHCA is now similar to that from nontraumatic cardiac arrest, which leaves no excuses for the resuscitation team to not resuscitate patients with TOHCA [2,8,18]. In addition to improving patient outcomes, our algorithm also provides a reasonable end point for resuscitation. If all evaluations and interventions are completed in the ED and no signs of circulation are detected, that might be the proper time to terminate resuscitation.

We anticipate that further improvements will be made to this algorithm. Resuscitative thoracotomy is a treatment option in cardiac arrest patients with penetrating injury [19,20]. However, no firm consensus on the role of resuscitative thoracotomy in cardiac arrest patients with blunt trauma has been reached. Our hospital did not attempt resuscitative thoracotomy in the ED because only 2.5% of patients sustained penetrating injury in this study and no trauma surgeons were involved in the resuscitation of patients with TOHCA. However, resuscitative thoracotomy should be integrated into a revised algorithm in the future. Additionally, in one study, targeted temperature management was shown to be beneficial in postresuscitative care for patients with nontraumatic out-of-hospital cardiac arrest [21]. However, hypothermia-related coagulopathy is a major concern for trauma patients, and hypothermia therapy is not indicated for patients with TOHCA [3]. For patients whose cause of death is not related to exsanguination but is instead due to-for instance-hanging, drowning, asphyxia, or electrocution, we considered including targeted temperature management in the standard postresuscitative care.

Limitations

This study had several limitations. First, because all the data were obtained from retrospective chart reviews, some of the patients’ prehospital data were lost. Despite this flaw, analysis of prehospital data revealed that prehospital interventions had little influence on patient outcomes, implying that the lost data might not have changed the results. Second, the data were derived from a single trauma center, which may not be representative of all patients with TOHCA. Nevertheless, the study hospital is a regional trauma center and has the most ED visits (120,000 annually) of all hospitals in the region. We believe that the collected data can represent the general situation of patients with TOHCA in Taiwan. Third, the algorithm is not a mandatory requirement, and not all EPs followed the protocol during resuscitation. However, we noticed that most EPs used this protocol after it was found to increase ROSC rates in patients with TOHCA. We believe that the algorithm will provide further benefits if it is adopted more broadly.

## Conclusions

We implemented an algorithm to improve the outcome of patients with TOHCA. This algorithm placed the highest priority on three major treatable causes of TOHCA: impedance of venous return, hypovolemia, and hypoxia. After implementing the algorithm, we discovered that rate of ROSC increased with the increasing implementation of ED interventions, especially the placement of a large-bore central venous catheter and blood transfusion. After achieving ROSC, higher ETCO2 values were correlated with a higher chance of hospital admission.
